# A 200 km suspected impact crater Kotuykanskaya near Popigai, Siberia, in the light of new gravity aspects from EIGEN 6C4, and other data

**DOI:** 10.1038/s41598-020-62998-6

**Published:** 2020-04-08

**Authors:** Jaroslav Klokočník, Jan Kostelecký, Aleš Bezděk, Gunther Kletetschka, Hana Staňková

**Affiliations:** 1 Astronomical Institute, Czech Academy of Sciences, CZ 251 65 Ondřejov, Fričova 298, Czech Republic; 2 Research Institute of Geodesy, Topography and Cartography, CZ 250 66 Zdiby 98, Prague, Czech Republic; 3 Faculty of Science, Charles University Prague, Czech Republic; 4 Faculty of Civil Engineering, Czech Technical University in Prague, CZ 166 29 Praha 6, Prague, Czech Republic; 5 Faculty of Mining and Geology, VSB-TU Ostrava, CZ 708 33, Ostrava, Czech Republic; 6 Geophysical Institute, University of Alaska Fairbanks, 903 N Koyukuk Drive, Fairbanks, AK, 39 USA

**Keywords:** Structural geology, Planetary science, Geomorphology, Geology

## Abstract

We provide arguments in favour of impact origin of a 200 km suspected impact crater Kotuykanskaya near Popigai, Siberia, Russia. We use the gravity aspects (gravity disturbances, the Marussi tensor of the second derivatives of the disturbing geopotential, the gravity invariants and their specific ratio, the strike angles and the virtual deformations), all derived from the combined static gravity field model EIGEN 6C4, with the ground resolution of about 10 km and a precision of about 10 milliGals. We also use the magnetic anomalies from the model EMAG2 and emphasize the evidence of much deeper sources in the suspected area, constraining the impact origin of this structure.

## Introduction

### Motivation, method, theory, and data

#### Motivation

The objective of this study is to use recently available high-resolution gravity aspects (descriptors) derived from the global Earth’s gravity field model EIGEN 6C4^1^ with the ground resolution of about 9 km, to provide an independent assessment of the existence of a suspected Kotuykanskaya impact crater(s) near the proven impact crater Popigai in Siberia, Russia.

It would be one of the largest impact craters on the Earth – with a diameter of ~200 km, about twice as large as Popigai, comparable to Chicxulub, so it is worthy to study it when we have available new gravity data and a new methodology (see comments below). Nevertheless, we have no detailed  geological data available, so we cannot provide any final decision.

For the given locality our aim is to apply the new available gravity aspects (derived from EIGEN 6C4), magnetic anomalies (EMAG v. 2) and surface topography (ETOPO 1).

#### Methodology

Classical gravity anomalies (or disturbances *Δg)* provide only limited information about the stress state of the rocks causing them. In order to broaden the potential information about the state of rocks we compute various gravity functions (the gravity aspects) of the disturbing gravitational potential expressed in the spherical harmonic expansion to a high degree and order (known as the geopotential coefficients or the Stokes parameters) in addition to the gravity anomalies *Δg*. The core of our method is in the use of various gravitational aspects, namely the components of the Marussi tensor **Γ** of the second derivatives *T*_*ij*_ of the disturbing potential, the gravity invariants *I*_1_ and *I*_2_, their specific ratio *I*, the strike angles *θ* and the virtual deformations *vd*. Each of these gravity aspect tells its own “story” about the density due to the causative body and in turn about the gravity signal generated.

#### Comments to theory

The theory underlying our methodology was described mainly in Pedersen & Rasmussen^2^ and Beiki & Pedersen^3^. The new contribution known as the *virtual deformation*, *vd*, comes from^4^. A more complete review of the theory (with examples) is in^5–7^. The examples devoted to the impact craters can be found in^5,8–10^ and cannot be repeated here owing to space reasons and selfplagiarism; thus, an extensive *Supplement* (with theory and examples) is added.

The Marussi tensor **Γ** having five independent components delivers more complex information than the gravity anomalies *Δg* alone; the location of the target body is related to the radial component *T*_*zz*_, the orientation and shape of the causative structure is characterised through the non-diagonal components *T*_*ij*_ of **Γ**^11^. The gravity invariants *I*_1_ and *I*_2_ act as non-linear filters augmenting the sources of substantial volumes; they help to divide larger density anomalies into individual units. Their specific ratio tells us about 2D structures. The strike angles *θ* inform us on how the observations (used to derive **Γ**) correlate with the main directions of the underground structure; these are the main direction(s) of **Γ**. Analogously to the tidal deformation, the virtual deformation *vd* describes the “tensions” (compression and dilatation) produced by the body in question; one can conceive such a deformation as being due to “erosion” caused solely by gravity (first described in^4^).

In the literature, many examples of the local use of usual gravity aspects (mostly *Δg*, sometimes *T*_*ij*_, seldom *I*_1_ and *I*_2_ or *θ*) can be found (see, e.g^3,11–13^.). Our methodology and various results covering different parts of the world can be found in^4,6–9,14–16^. The values of all the gravity aspects were obtained by using the software by B. Bucha^17^ and by our own programs.

Colour figures presented here have various non-linear scales to emphasize specific features and details. The gravity disturbances are given in milligals [*mGal*], the second order derivatives are in Eötvös [*E*]. Recall that 1 *mGal* = 10^−5^ *ms*^−2^, 1*E* ≡ 1 Eötvös = 10^−9^*s*^−2^. The strike angles *θ* in all figures are expressed in degrees with respect to the local meridian (north-south direction): the red arrows indicate its direction to the west and those in blue to the east. The invariants have units [*s*^−4^] and [*s*^−6^]. The virtual deformations (*vd*) are shown in blue colour, where compression takes place and in red where dilatation occurs. The magnetic anomalies are in nanotesla [*nT*].

### Data

#### Gravity

The EIGEN 6C4 (*E*uropean *I*mproved *G*ravity model of the *E*arth by *N*ew techniques)^1^ is a global, static, combined, comprehensive and detailed gravity field model including gradiometry from the whole GOCE mission (*G*ravity field and steady-state *O*cean *C*irculation *E*xplorer, ESA, in orbit in 2009–2013). The important fact is that EIGEN 6C4 is a better and higher resolution gravity model in a comparison with all its predecessors. Its terrestrial data (very important component of the model from the high-resolution part) came from the older EGM 2008 gravity model and other sources (like satellite altimetry). Its worldwide resolution is 5 × 5 arcmin, which is about 10 km on the ground and its precision, expressed as a typical standard deviation in the gravity anomalies, is about 10 mGal. The quality of EIGEN 6C4 is not given only by its resolution and precision, but also by a global and nearly regular coverage (excluding small polar gaps) achieved by the five years of GOCE gradiometry (http://www.esa.int/GOCE). In this study, we solely make use of EIGEN 6C4.

#### Impact structures inventories

The *Earth Impact Database* is a database of confirmed, proved impact structures, now with nearly 200 objects, see http://www.passc.net/EarthImpactDatabase/index.html, developed and maintained by the Planetary and Space Science Centre, University of New Brunswick, Canada. Unconfirmed impact craters were summarized by Rajmon in the previous SEIS (Suspected Earth Impact Sites) catalogue, now in^18^. Kotuykanskaya objects are not listed there. Another inventory has been worked out by Russian authors, http://labmpg.sscc.ru/impact/index1.html; see Mikheeva^19^. It contains 260 proven, 260 probable, 2190 potential, 630 questionable and 45 discredited impact structures. A few of them are located near the well-known, confirmed Popigai (I) impact crater. Popigai itself might be in fact a multiple crater^8^, which is, however, not yet confirmed (but it was accepted and applied in^20^). Kotuykanskaya objects are listed there (using gravity anomalies from old gravity field model EGM 2008^8^).

#### Magnetic anomalies

We add magnetic anomalies from the EMAG2 project, which is a global 2-arc-minute resolution grid of the anomaly of the magnetic intensity at an altitude of 4 km above mean sea level, compiled from satellite, marine, aeromagnetic and ground magnetic surveys^21^. For the area of Russia, the authors mention the resolution of magnetic anomalies to be around 5 km. While data are provided in 2 arcmin resolution, the resolution of magnetic signatures is dictated by the distance from the source that exceeds the altitude of the survey^22–25^. Wherever available, the original shipborne and airborne data replaced the precompiled oceanic magnetic grids and interpolation between sparse track lines in the oceans was improved by directional gridding and extrapolation, based on an oceanic crustal age model^21^.

#### Surface topography

**Figure 1:**
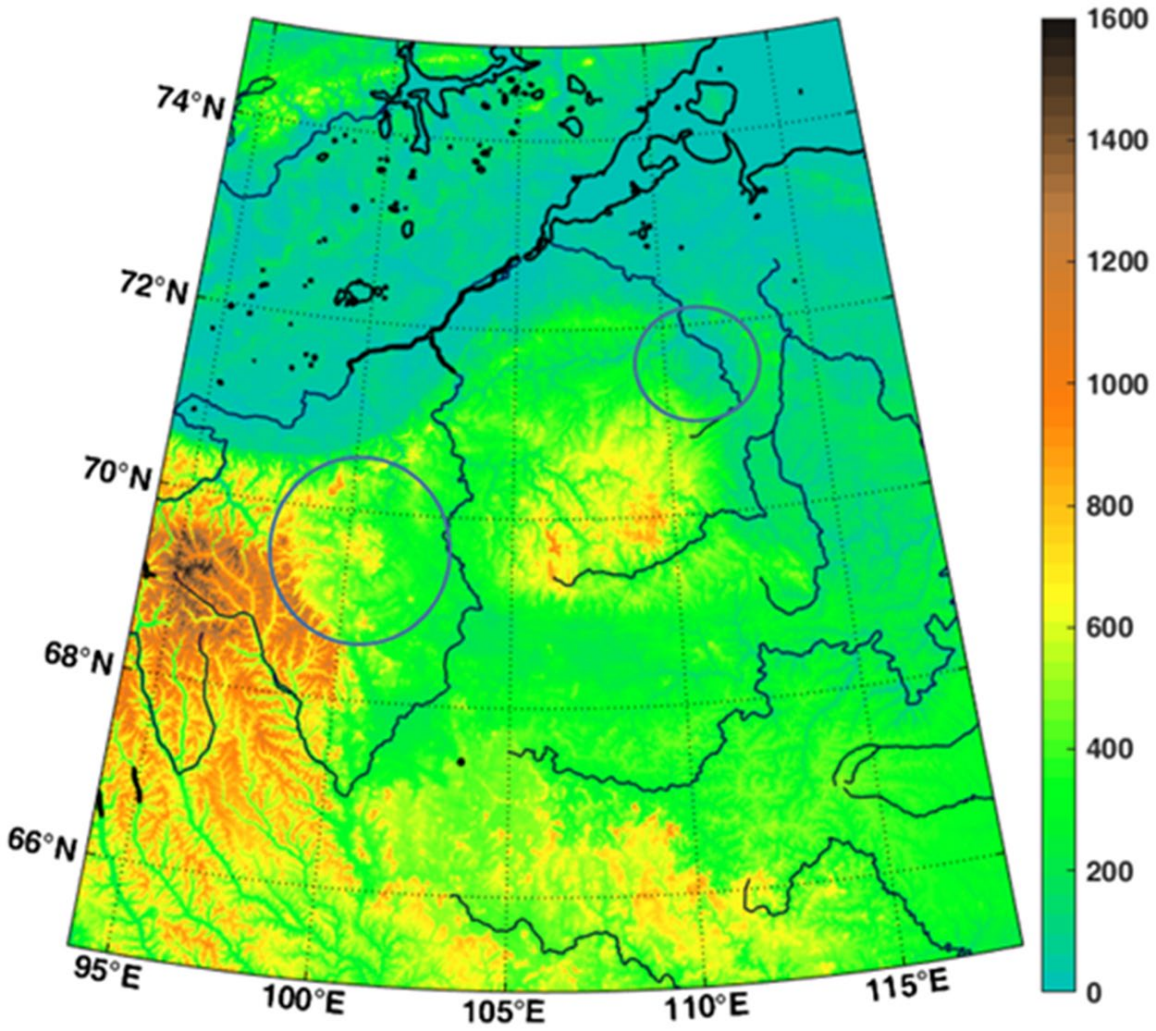
ETOPO 1 topography [m] of the Popigai and Kotuykanskaya area, Siberia.

Topography model ETOPO 1^26^ is a global 1-arcmin (cell size) relief model of the Earth surface that integrates land topography and ocean bathymetry from huge number of satellite measurements. Its precision is about 10–15 m in heights (but not everywhere). Topography from ETOPO 1 model for our area of interest is plotted in Fig. 1a. It is not in a conflict with our other results (see below) but, in a contrast to Popigai itself, the existence of a hole after the impact is not shown clearly. It depends on subsequent effects of geologic forces acting on the spot, here probably mainly water erosion, and on resolution of the topography model in the given area. The surface topography for Kotuykanskaya II is available also from a local Russian remote sensing aircraft material^19^ but the data themselves are not accessible. Topography from ETOPO1 for the area is in Fig. 1.

#### Geological map

**Figure 2:**
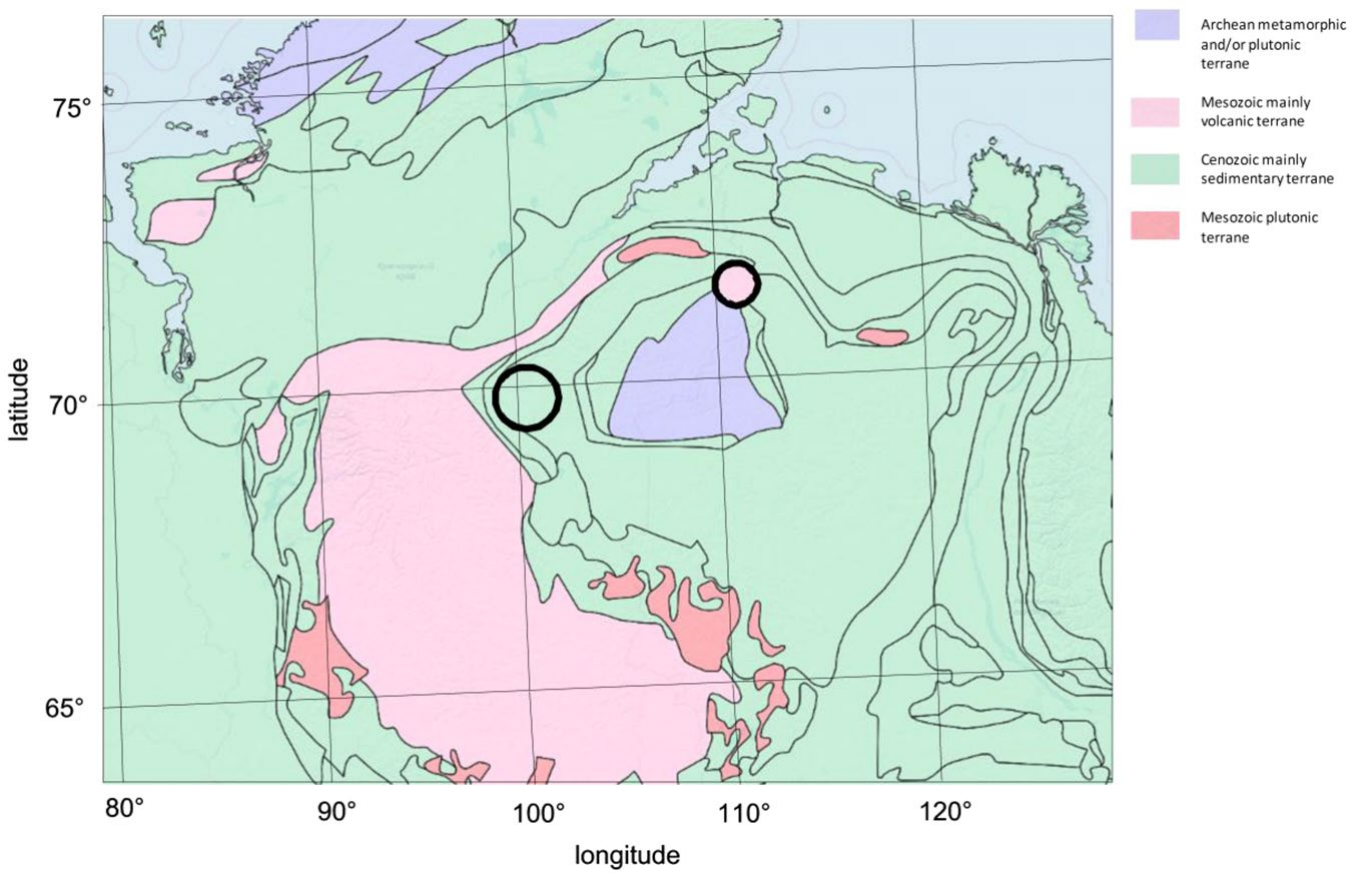
Reproduced from General Geologic map of the world, reproduced 1:1 from Geological Survey of Canada, Open File Report 2915. Interpretation is in Section *Discussion*.

A general geologic map of the world is available from the Geological Survey of Canada at approximately 1:35,000,000 scale. We have the area of our interest in Fig. 2.

#### *In situ* data

Such geological data as shocked quartz and other impact related grains, shatter cones, coesit, stishovite, diamonds (see the case of Popigai) and other signs of the impact origin are not available, at least in open literature. Very probably such data are not and will not be available owing to the specific locality in Siberia. We have to work with what is available and thus be conservative as for our conclusions.

## Results and discussion

### Gravity signal expected for impact craters

What gravity signal is expected (according to our experience) for the proven impact crater? It is expected that *Δg* and *T*_*zz*_ would be negative inside the crater (in the “hole” on open air or covered by less-dense newer sediments), changing positive and negative values for the respective rims around the crater and in space between them. The gravity invariants have extreme values inside and around the crater itself and they are significantly concentrated to “spots” (blotches?), creating isolated extremes in the rim(s) along the crater’s ring. The strike angles *θ* usually show a certain degree of orientation (they are “combed”) into one prevailing direction inside, around and near the crater, which is not true in an arbitrary location – the strike angles are generally chaotic (“dishevelled”). But there are places with such a specific orientation, lakes, paleolakes, ground water, deep river valleys, rifts, craters, oil/gas deposits, etc^15^. The virtual deformations *vd* inside the crater indicates a compression surrounded by a dilatation pattern. These gravity aspects are typical for all sufficiently large impact structures where we are can apply our method (otherwise being limited by the present-day available gravity field data resolution ~10 km).

### Results for Kotuykanskaya

**Figure 3:**
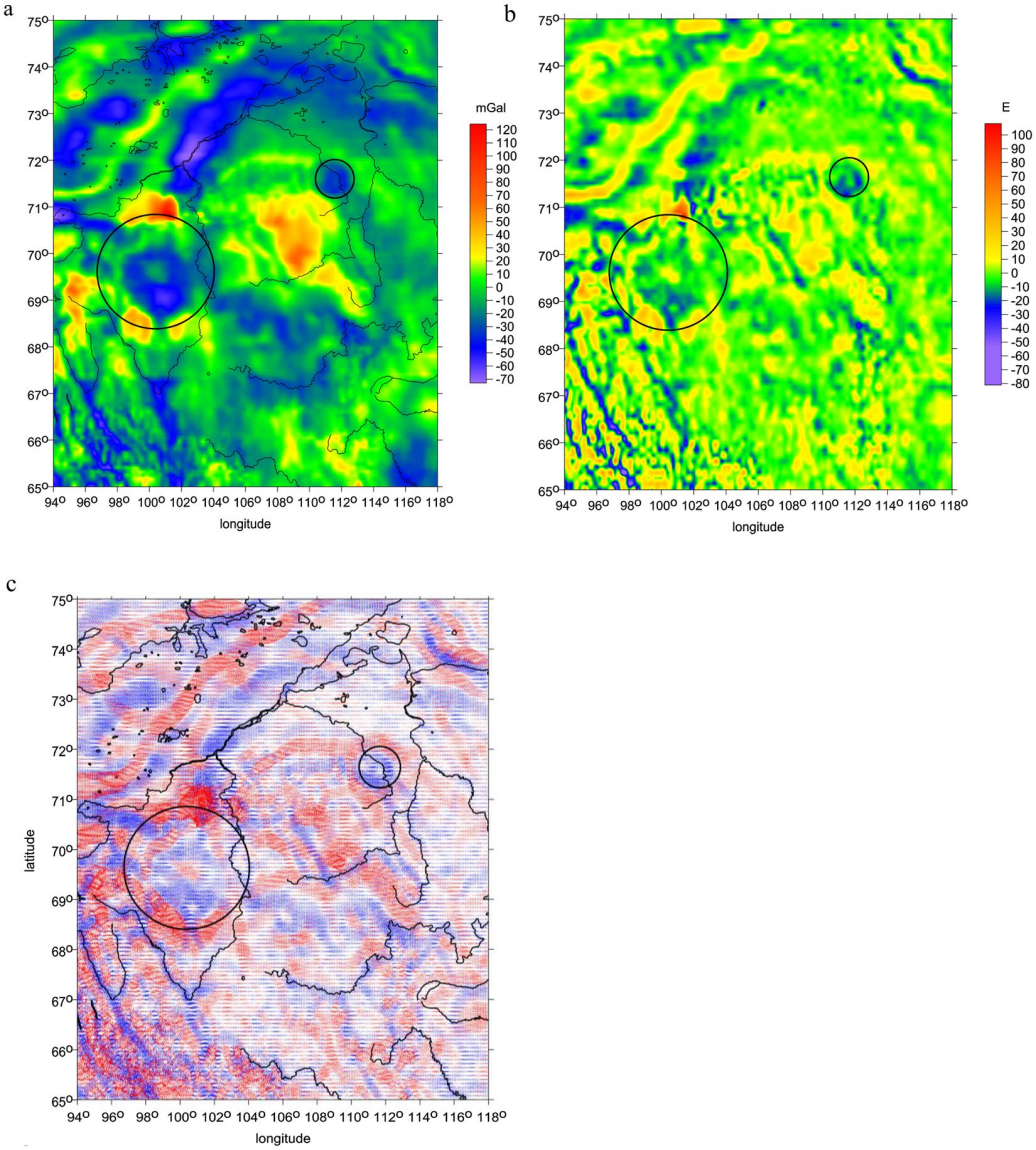
(**a–c**) The gravity disturbances *Δg* [mGal], the radial second order derivative *T*_*zz*_ [E], and the virtual deformations *vd* [-] for the Popigai and Kotuykanskaya area with EIGEN 6C4; blue for compression, red for dilatation. Compare a relatively regular positive belt around Popigai with positive but fragmented belt around Kotuykanskaya, with denudated terrain and signal on eastern side.

**Figure 4:**
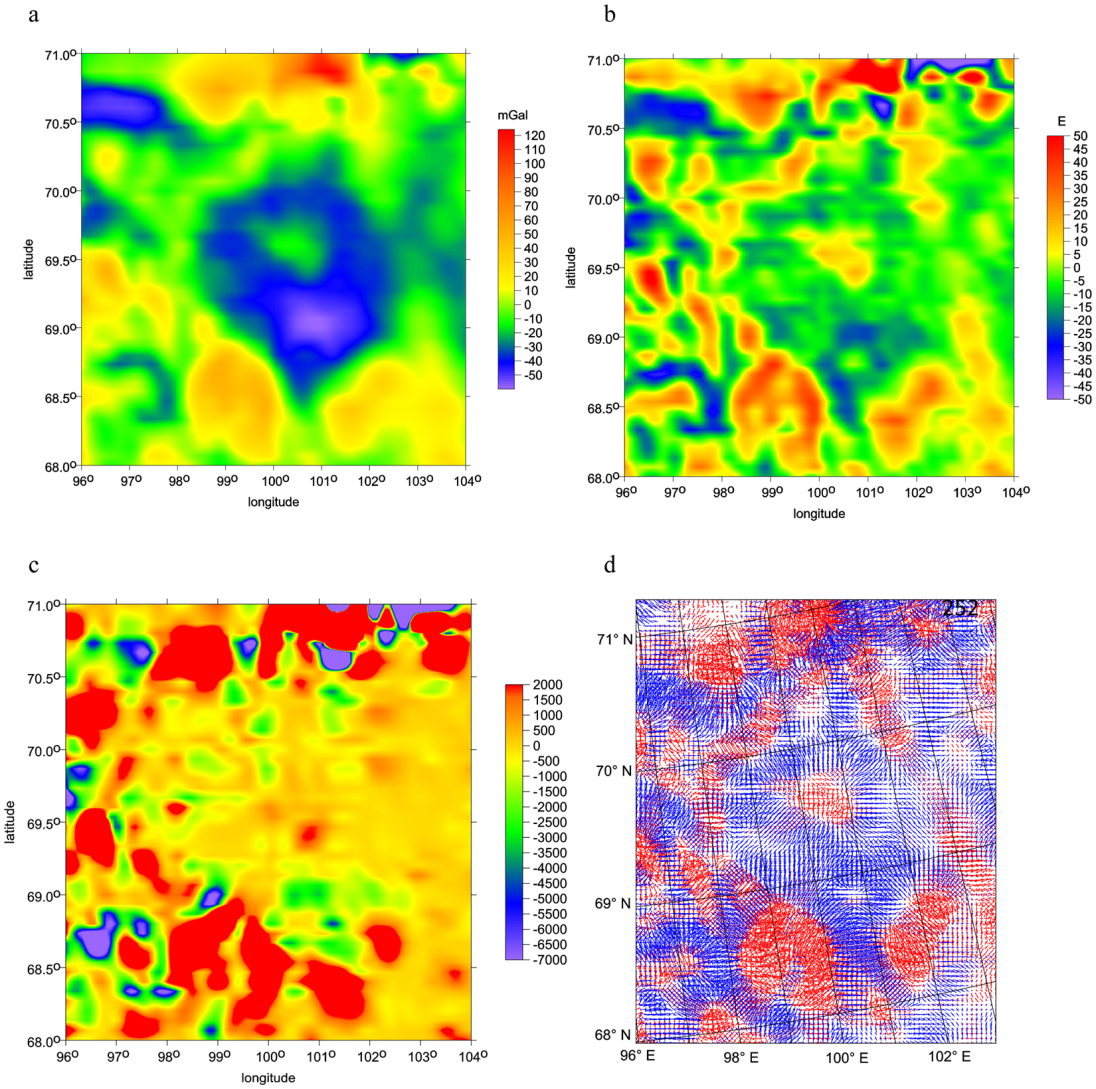
(**a–d**) A zoom to Fig. 3. The gravity disturbances *Δg* [mGal], the radial second order derivative *T*_*zz*_ [E], the gravity invariant *I*_2_ [s^−6^], and the virtual deformations *vd* [-] (red for dilatation, blue for compression) in the Kotuykanskaya II suspected impact crater, computed with EIGEN 6C4. Owing to high latitude, to get reliably plotted orientation of *vd* “vectors”, we had to use for this gravity aspect the polar projection.

First, we refer to the gravity anomalies derived from an older gravity model EGM 2008^8^, as amended by circles around the impact and suspected impact structures in^19^. Then, in the same area, with a focus to Kotuykanskaya II, we present a choice of the gravity aspects, computed from EIGEN 6C4 (Fig. 3a–c, 4a–d).

**Figure 5:**
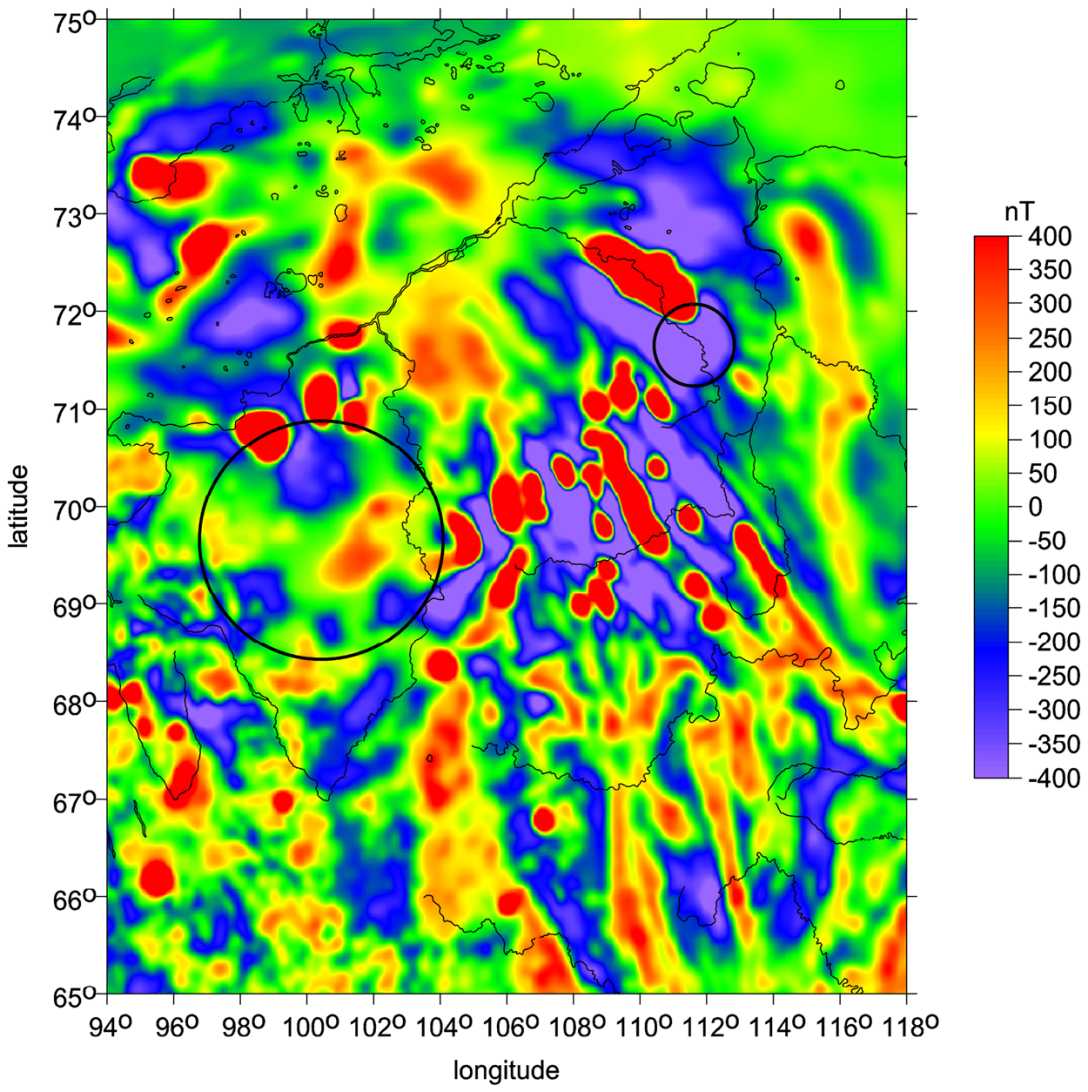
Magnetic anomalies [nT] for the Popigai and Kotuykanskaya area according to EMAG2. Note that the magnetic anomalies have sharp contrast and larger magnitudes outside the suspected crater boundary while inside the crater boundary, anomalies have smaller magnitudes and are blurred.

Finally, we add magnetic anomalies (Fig. 5) from the EMAG2^21^. Both structures, the Popigai proven impact crater as well as the Kotuykanskaya II putative impact crater, exhibit a decline in magnitude (more strongly for Kotuykanskaya II) in the magnetic anomalies when moving towards the centre of the proposed crater (the question is their interpretation, see *Discussion*).

## Discussion

### Gravity field

Now we discuss our “gravity observations” for the Popigai and Kotuykanskaya area, first for both crater together (Fig. 3a–c), and then in zooms for Kotuykanskaya II (Fig. 4a–d). The gravity disturbances *Δg* (Fig. 3a) and the radial component *T*_*zz*_ (Fig. 3b) are negative and circular inside the crater or putative crater and Kotuykanskaya II clearly shows a large central peak, namely in the second derivative (which is expected, because the second derivative is more sensitive to shallower density variations). The craters Popigai II, III…, predicted in^8^, are lined up in NW-SE direction from the most intensive Popigai (I) on NW, well visible in Fig. 3a,b, their right upper corner.

Kotuykanskaya has in its southern part a crater inside the main crater also with a central peak well shown mainly by *T*_*zz*_. The rim around Popigai is fragmented (by erosion?). The rim (or rims?) around Kotuykanskaya are very fragmented and partly missing on the eastern side (the same is true for the surface topography, Fig. 1). The *vd* show dilatation at the rims and compression inside the craters (Fig. 3c).

The zooms for Kotuykanskaya (Fig. 4a-b) repeat *Δg* and *T*_*zz*_ with more details about the inner crater, central peaks and fragmented ring(s). They show the smaller crater inside the crater also with a central peak. The *I*_2_ values in Fig. 4c concentrate power into isolated peaks in the ring, completely missing on east side. The *vd* in Fig. 4d exhibit, as also expected from previous experience with other craters, compression inside the crater and dilatation around it in the ring. The central peaks are denoted in all Fig. 4. These are not artefacts but real features; the resolution is still sufficient.

All these observations support the hypothesis about the impact origin of Kotuykanskaya II and Kotuykanskaya I. We accept of course that the gravity-based results are not unique. For this reason we discuss also “magnetic results” for the Popigai and Kotuykanskaya area (Fig. 5) trying to strengthen or to challenge our results with the gravity aspects.

### Magnetic field

Magnetic anomalies (Fig. 5) reflect two types of magnetization, induced and remanent^27–30^. While the general expression of magnetic anomalies on Earth is driven by induced magnetization due to main dipolar geomagnetic field areas containing an even moderate amount of titanohematite and/or hematite, reflecting the oxidation of the crust, are dominated by remanent magnetization^25,28,31–34^. When the crust is impacted by meteorite whose mass is such that the atmosphere has negligible effect on the slowing down the impactor, the speed of the impact has tens of km/s. The resulting impact and explosion create a shock pressure exposure of the crust that decays exponentially from the impact epicentre^35,36^. Such pressure exposure necessary demagnetizes the crust. As the shock wave compresses the magnetic carriers, it randomizes the overall magnetic energy (on shock wave passing, the carrier is behaving like superparamagnetic grain when magnetic moments are fluctuating chaotically^24,31^). Different minerals have different sensitivities to the impact demagnetization^36^. In general, the multidomain magnetite is most sensitive, followed by single domain magnetite, multidomain pyrrhotite, and multidomain hematite. The most resistant is exsolved titanohematite, that can be abundant in the Earth’s crust^27,37^. The crustal rocks in this area are exposed to the main component of the dipolar field. As a result, there are two components of magnetization that form magnetic anomalies. Positive anomalies are in the direction of the main dipolar field. First look at Popigai in Fig. 5. There are three clusters of positive (red) anomalies around the crater. These are due to iron rich bodies with inherent induced magnetization. Note that Popigai is centred on the edge of the large positive anomaly to the north, cluster of positive anomalies to the south west and small positive anomaly to the south east. The space in between these three anomalies is filled with negative anomaly centred over the crater area. This interruption of the induced magnetic signature we interpret is due to demagnetization of the impact. The demagnetization penetrates down at least the crater’s radius.

The Kotuykanskaya structure shows that around the edges of the presumed crater are well defined induced magnetic anomalies (in red). Since the crater is 200 km wide the demagnetization penetrates at least to the same depth of 200 km. Such a process is connected with plasma generation that is capable to penetrate and permanently magnetize the deep previously demagnetized material resulting in four to five blurred magnetic anomalies similar like in the case of the Chicxulub impact crater^38^.

The magnetic map shows details of magnetic field over the suspected impact structures. Note the contrast of sharpness of magnetic anomalies around the Kotuykanskaya II structure and inside the structure. This difference separates the magnetic sources that are close to the surface that shows the sharp magnetic boundaries from sources that are deep-seated and whose boundaries are not as sharp. Positive anomalies (red colour) show induced magnetization, locations where there is likely a large amount of magnetite, the main carrier of induced magnetization^27,34^. Geologic units containing these sources are distributed not only in the north from the crater but also southward and eastward from the suspected crater. The centre of the crater where we see the diffused magnetic boundaries have been demagnetized to a depth of several tens of kilometres and left magnetization only of magnetic sources that are deep-seated. This location correlates well with the location of the suspected crater and leaves an evidence of impact demagnetization process^35,36^.

The magnetic map shows similar signs of demagnetization over the Popigai structure. The structure is bounded by four distinct positive anomalies, the largest from the north and two moderate southwest but also one from the southeast. The large negative field detected over the centre is consistent with impact demagnetization similarly like Kotuykanskaya and supports the impact origin.

Assuming geology from the Geological Survey of Canada (back to Fig. 2), the position of the proposed crater indicates the Mesozoic age, while the age of Popigai is of the Cenozoic age. An impact crater that happen to Mesozoic sediment creates a hole in it and exposes the underlying Mesozoic geology. There is an evidence that the impact created an actual topographic relief^39^. The black lines are Cenozoic geologic contacts, and they interestingly round around the Kotuykanskaya structure. This would be an indication that the Cenozoic sedimentary terrain were filling the dimple made by the cratering process in Mesozoic and some of these Cenozoic sedimentary units were subsequently eroding away leaving the exposures of Cenozoic geological contacts. The structure can be a Mesozoic event, from about 251 to 66 million years ago. While we are aware that the age estimates are not precise, the geology map in Fig. 2 and the relationship of the units offers the most simple and straightforward interpretation of the Kotuykanskaya II crater age.

## Conclusion

While there is no direct evidence of shock in terms of shatter cones, planar deformation features, high pressure minerals, etc., we provide evidence in terms of new gravity and magnetic data and new methodology that at least Kotuykanskaya II, the suspected impact structure near Popigai, Siberia, is likely created by impact process. Kotuykanskaya would be one of the largest impact craters on the Earth – with a diameter about 200 km, so further studies are recommended.

## Data Availability

The input data from the models global models EIGEN 6C4, EMAG v2, and ETOPO 1 are publicly available (see the list of reference below). The outputs (the files to plot our figures) are available upon request from the authors.
